# A Denoising Method for Fiber Optic Gyroscope Based on Variational Mode Decomposition and Beetle Swarm Antenna Search Algorithm

**DOI:** 10.3390/e22070765

**Published:** 2020-07-13

**Authors:** Pengfei Wang, Yanbin Gao, Menghao Wu, Fan Zhang, Guangchun Li, Chao Qin

**Affiliations:** Collage of Automation, Harbin Engineering University, Harbin 150001, China; hbforwpf@hrbeu.edu.cn (P.W.); wumenghao@hrbeu.edu.cn (M.W.); zhangfan41@hrbeu.edu.cn (F.Z.); lgc_67@hrbeu.edu.cn (G.L.); max_qinchao@hrbeu.edu.cn (C.Q.)

**Keywords:** beetle swarm antenna search algorithm, permutation entropy, variational mode decomposition, fiber optic gyroscope, signal denoising

## Abstract

Fiber optic gyroscope (FOG) is one of the important components of Inertial Navigation Systems (INS). In order to improve the accuracy of the INS, it is necessary to suppress the random error of the FOG signal. In this paper, a variational mode decomposition (VMD) denoising method based on beetle swarm antenna search (BSAS) algorithm is proposed to reduce the noise in FOG signal. Firstly, the BSAS algorithm is introduced in detail. Then, the permutation entropy of the band-limited intrinsic mode functions (BLIMFs) is taken as the optimization index, and two key parameters of VMD algorithm, including decomposition mode number *K* and quadratic penalty factor α, are optimized by using the BSAS algorithm. Next, a new method based on Hausdorff distance (HD) between the probability density function (PDF) of all BLIMFs and that of the original signal is proposed in this paper to determine the relevant modes. Finally, the selected BLIMF components are reconstructed to get the denoised signal. In addition, the simulation results show that the proposed scheme is better than the existing schemes in terms of noise reduction performance. Two experiments further demonstrate the priority of the proposed scheme in the FOG noise reduction compared with other schemes.

## 1. Introduction

Inertial navigation is an autonomous navigation method. Because of its uniqueness, the inertial navigation system (INS) is widely used in many fields [1,2,3]. Fiber optic gyroscope (FOG) is one of the most common hardware for inertial system, it has the advantages of high measurement accuracy and large measurement range [4,5]. However, due to the influence of external environment and signal sampling conditions, the output signal of FOG often contains a lot of random noise [6,7]. It will greatly affect the accuracy of INS. Therefore, it is necessary to suppress the random noise of FOG signal.

In general, there are three different ways to deal with random noise of FOG signal. The first one is to establish autoregressive moving average (ARMA) model based on the random noise sequence, and then the model is optimized by filtering methods [8,9]. Although this method performs well under the premise of accurate noise model is established, it is difficult to get accurate random noise model in practical application, so the noise reduction effect will be greatly affected [10,11].

Using filtering algorithm to reduce noise directly is the second common method. Narasimhappa et al. proposed a robust adaptive Kalman filtering algorithm to filter the FOG signal directly [12]. In this method, a new covariance matrix of measurement noise is designed to improve the filtering effect of the adaptive Kalman filtering algorithm. He et al. proposed a hybrid algorithm which combining the advantages of adaptive chirp mode pursuit (ACMP) algorithm and adaptive multiscale Savitzky–Golay filter (AMSGF) algorithm [13]. In this method, the ACMP algorithm is uesd to denoise the FOG signal roughly, and then the AMSGF algorithm is utilized to filter it further. All in all, although the second common method does not need to establish the mathematical model of noise sequence, the effect of the denoising is not ideal because of the relatively simple denoising process.

The third way to deal with FOG signal is to decompose the original signal first, and then analyze the decomposed components. The common methods for decomposing signal include wavelet transform (WT) [14] and empirical mode decomposition (EMD) [15]. WT can decompose the signal into components of different frequency bands. Zhang et al. put forward an improved wavelet threshold transform to process the random noise of FOG signal [16]. The experimental results show that this method can get lower mean square error. Ma et al. applied the optimized WT filter to the magnetic levitation gyroscope (GAT) and successfully reduced the vibration noise in actual use [17]. The WT method can effectively reduce the random noise in FOG signal, but this method needs to preset wavelet basis function and decomposition level, so the adaptability is not good. EMD can decompose the original signal into a series of intrinsic mode functions (IMFs) by analyzing the local characteristic time scale of the signal. Liu et al. developed a denoising method combining EMD and interval threshold methods to suppress the random error of FOG signal successfully [18]. Wang et al. presented a new method which combined EMD and recursive least square (RLS) method to reduce the noise of FOG signal. Firstly, EMD is used to decompose the noisy signal, then RLS algorithm is used to filter the selected IMFs [19]. Different from the WT method, the EMD algorithm can decompose different data adaptively, but the disadvantages of mode mixing and end effect can not be ignored.

Variational mode decomposition (VMD) is a new signal decomposition method proposed in recent years [20]. It overcomes the problem of mode mixing in the EMD method and can deal with non recursive signals better. Wu et al. employed VMD to decompose the FOG signal, and then the decomposed signal was filtered based on the generalized morphological filter (CGMF). They proved the feasibility of the proposed scheme through experiments [21]. Zhang et al. first decomposed the gyroscope signal by utilizing VMD, then filtered the component signal by the adaptive Sigmoid function-based tracking differentiator (STD), and finally realized an efficient denoising [22]. Although VMD algorithm performs well in signal decomposition, its performance is affected by two parameters, namely decomposition mode number *K* and quadratic penalty factor α. A traditional trial and error method that depends on experience will lead to an unreliable result and will waste a lot of time. It will greatly limits the performance of the VMD algorithm. Besides, the selection of relevant modes is also a problem.

In order to solve the difficulties of the VMD algorithm in processing signal noise, a new method based on the beetle swarm antenna search (BSAS) algorithm and the VMD algorithm is proposed in this paper. The other five section of this paper are arranged as follows. In Section 2, the BSAS algorithm, which is a new intelligent optimization algorithm, is described in detail. In addition, VMD algorithm will also be explained in this section. The denoising procedures for the FOG signal based on the proposed method will be introduced in Section 3. In Section 4, through a simulation study, the performance of the proposed scheme is compared with the other three common schemes, and the superiority of the proposed scheme is confirmed by analyzing the simulation results. Next, in Section 5, static and dynamic rotation test experiments based on FOG further prove the feasibility and effectiveness of the proposed scheme. Finally, in the last section, the conclusion of this paper is presented, and the future work is prospected.

## 2. Theoretical Background

### 2.1. Beetle Swarm Antenna Search Algorithm

The beetle swarm antenna search (BSAS) algorithm is a new intelligent optimization algorithm which was proposed in 2018 [23,24]. It is a variant algorithm based on a beetle antennae search (BAS) algorithm which was proposed in 2017 [25]. The BAS algorithm optimizes the optimization index by imitating the predatory behavior of the beetle. When the beetle is preying, the two antennae on the left and right sides of its head will receive food odour from two directions respectively. According to the odour intensity in two directions, the beetle will choose to approach the direction with stronger odour intensity.

The BSAS algorithm is an improved algorithm of BAS. It expands the number of beetles from a single one in the BAS algorithm to a beetle group, which greatly improves the optimization ability and stability of the algorithm. Besides, compared with the BAS algorithm, the BSAS algorithm adds some additional parameters and improves the step size update strategy, which greatly improves the ability to solve complex problems.

The intensity of food odor can be expressed by the function to be optimized, (i.e., the fitness function), and then the biological behavior expressed by BSAS algorithm can be expressed by mathematical formula. The search direction of each beetle in the beetle group is randomly initialized at each search, as shown in Equation (1).

(1) $$\vec{d_{iri}}=\frac{rnd{\left(n,1\right)}_{i}}{{∥rnd{\left(n,1\right)}_{i}∥}_{2}},$$

where *i* is the currently selected beetle and *n* is the dimension of the variable to be optimized. rnd(·) denotes random function which can generate a random direction $d_{ir}$. The two antennae used to receive odor intensity can be expressed as follows:

(2) $$\left\{\begin{array}{c} x_{ri}^{k}=x_{i}^{k}+d^{k}{\vec{d}}_{iri}\;\\ x_{li}^{k}=x_{i}^{k}-d^{k}{\vec{d}}_{iri} \end{array}\right.$$

In Equation (2), *k* represents the current iteration times, and $x_{li}$ and $x_{ri}$ represent the left and right antennae of the *i*th beetle. $d^{k}$ is sensing distance which denotes the length between the antennae and the head of beetle. $d^{k}$ can be expressed as Equation (3), where $d_{0}$ is the minimum resolution of sensing distance, it needs to be set in advance. $c_{d}$ is the decay coefficient of sensing distance.

(3) $$\left\{\begin{array}{c} d^{k}=c_{d}d^{k-1}+d_{0}\;\\ c_{d}\in \left(0,1\right) \end{array}\right..$$

Based on the concept of beetle group in BSAS algorithm, the position $x_{i}^{k}$ of each beetle in the beetle group can be obtained as shown in Equation (4)

(4) $$x_{i}^{k}=x_{i}^{k-1}+s^{k}\cdot {\vec{d}}_{iri}\cdot sign\left(f\left(x_{ri}^{k-1}\right)-f\left(x_{li}^{k-1}\right)\right),$$

where f(·) represents the odor intensity (i.e., the fitness function). sign is symbolic function, which can choose a more ideal optimization direction for the next iteration. $s^{k}$ is search step size, as shown in Equation (5), $s_{0}$ is the minimum resolution of the search step size, and $c_{s}$ is the decay coefficient of the search step size.

(5) $$\left\{\begin{array}{c} s^{k}=c_{s}s^{k-1}+s_{0}\;\\ c_{s}\in \left(0,1\right) \end{array}\right..$$

In addition, compared with the BAS algorithm, two probability constants $p_{st}$ and $p_{pos}$, which are between 0 and 1, are added to the BSAS algorithm. Where $p_{st}$ is used to control the step size update. It means that when no better position is found, the probability that the step size will be updated in the next iteration is $p_{st}$, and the probability that it will not be updated is $\left(1-p_{st}\right)$. By setting the $p_{st}$ parameter, the search step size will no longer be unconditionally updated and reduced, thus it can improve the ability of global optimization. Moreover, in order to ensure the efficiency of the algorithm and avoid the bad results caused by improper $p_{st}$ parameter setting, the BSAS algorithm proposes the $n_{st}$ parameter. $n_{st}$ represents the maximum number of invalid searches by using the same step size. When the cumulative number exceeds $n_{st}$, in the next iteration, the search step size will be forced to update.

Similar to $p_{st}$, $p_{pos}$ is used to control beetle position update. It means that in each iteration, if *M* beetles in the beetle group find a better position, then in the next iteration, the probability of beetle being updated to the best position is $p_{pos}$. On the contrary, the probability that the beetle will be updated to a better position rather than the best position is $\left(1-p_{pos}\right)$. By setting $p_{pos}$ parameter, the disturbance of local optimal value can be effectively reduced, and the ability of global optimization can be improved.

In summary, combined with the mathematical expression and biological characteristics of BSAS algorithm, the pseudocode of BSAS algorithm is shown in Algorithm 1.
**Algorithm 1** BSAS algorithm**Input:** Define the fitness function f(·). Set the optimization variable *x* to be optimized and determine its dimension *n*.**Output:**$x_{best}$ and $f_{best}$. 1: **Initialize:**   Initialize the position of beetle group $x_{i}^{0}$ and probability constant $p_{pos}$;   Initialize the search step size $s^{0}$ and decay coefficient $c_{s}$;   Initialize probability constant $p_{st}$ and set parameter $n_{st}$;   Initialize the sensing distance $d^{0}$ and decay coefficient $c_{d}$;   Initialize the maximum number of iterations $k_{max}$;   Set initial optimization results for $x_{best}$ and $f_{best}$. 2: **if**
$k<=k_{max}$
**then** 3:    Generate random directions ${\vec{d}}_{iri}$ for each beetle by Equation (1). 4:    Calculate the antennae position $x_{li}^{k}$ and $x_{ri}^{k}$ of each beetle by Equation (2). 5:    Calculate the position of each beetle $x_{i}^{k}$ by Equation (4), and calculate the fitness function value $f(x_{i}^{k})$. 6:    Compare all $f(x_{i}^{k})$ and $f_{best}$ in this iteration. 7:    **if** ∃$f\left(x_{i}^{k}\right)<f_{best}$
**then** 8:        **if**
$rand\left(1\right)=a<p_{pos}$
**then** 9:           $x_{best}$ =$argmin(f\left(x_{i}^{k}\right))$ 10:           $f_{best}$ =$min(f\left(x_{i}^{k}\right))$ 11:        **else** 12:           $x_{best}$ =$sample(x_{i}^{k})$ 13:           $f_{best}$ =$\left(f\left(x_{sample}^{k}\right)\right)$ 14:        **end if** 15:    **else** 16:        **if**
$rand\left(1\right)=b<p_{st}\left|\right|i>n_{st}$
**then** 17:           Search step $s^{k}$ is updated by Equation (5). 18:           Sensing distance $d^{k}$ is updated by Equation (3). 19:        **else** 20:           i=i+1 21:        **end if** 22:    **end if** 23:    k=k+1 24:**end if**

Compared with the BAS algorithm, the BSAS algorithm has greatly improved its optimization and stability ability by increasing the number of beetles. Furthermore, the BSAS algorithm introduces additional parameters in the step size strategy and position update strategy, which makes it more likely to find the global optimal value. Because of the advantages mentioned above, the BSAS algorithm is widely used in many fields [26,27,28].

### 2.2. Variational Mode Decomposition Algorithm

Dragomiretskiy et al. proposed a variational mode decomposition algorithm (VMD) in 2014, which can decompose the signal into *K* band-limited intrinsic mode functions (BLIMFs) [29]. The center frequency of each modal component is $\omega_{k}$.

Firstly, the estimation of the center frequency and bandwidth for BLIMF components is transformed into a variational problem with constraints by using VMD algorithm. Then, by introducing penalty factor and Lagrange function, the problem with constraint condition is transformed into the problem without constraint condition. Finally, the alternative multiplier algorithm is used to get the optimal solution. The VMD algorithm can be regarded as an improved algorithm of Wiener filter [30]. It effectively solves the problem of modal aliasing in the EMD algorithm [31]. The specific VMD algorithm can be expressed as follows:

To begin with, the signal x(t) is decomposed into *K* BLIMF components $u_{k}\left(t\right)$, k=1,2,⋯,K, as shown in Equations (6) and (7).

(6) $$x\left(t\right)=\sum_{i=1}^{K}u_{k}\left(t\right)$$

(7) $$u_{k}\left(t\right)=A_{k}\left(t\right)\cos\left(\varphi_{k}\left(t\right)\right).$$

The instantaneous frequency $\omega_{k}\left(t\right)$ of $u_{k}\left(t\right)$ is presented as Equation (8):

(8) $$\omega_{k}\left(t\right)=\frac{d\varphi_{k}\left(t\right)}{dt}.$$

After that, the Hilbert transform is applied to each mode component, and the frequency spectrum of each BLIMF component is modulated to the frequency band with the center frequency $\omega_{k}$, as shown in Equation (9):

(9) $$\left[\delta \left(t\right)+\frac{j}{\pi t}\right]*u_{k}\left(t\right)\cdot e^{-j\omega_{k}t},$$

where δ(t) is the Dirac distribution, and $\omega_{k}$ is the center frequency. * represents symbol of convolution operation. Next, the Gaussian smoothness of the transformed signal can be used to estimate the bandwidth of each component. The variational problem with constraints is given as Equation (10):

(10) $$\begin{array}{cc} \; & \min_{\left\{u_{k}\right\},\left\{\omega_{k}\right\}}\left\{\sum_{k=1}^{K}{∥\partial_{t}\left[\left(\delta \left(t\right)+\frac{j}{\pi t}\right)*u_{k}\left(t\right)\right]\cdot e^{-j\omega_{k}t}∥}_{2}^{2}\right\}\\ \; & \;s.t.\;\sum_{k=1}^{K}u_{k}=x\left(t\right). \end{array}$$

By introducing quadratic penalty factor α and Lagrange multiplication operator λ(t), the constrained variational problem can be transformed into unconstrained variational problem. It should be noted that the augmented Lagrangian is expressed as Equation (11)

(11) $$\begin{array}{cc} L\left(\left\{u_{k}\right\},\left\{\omega_{k}\right\},\lambda \right)= & \alpha \sum_{k=1}^{K}{∥\partial_{t}\left[\left(\delta \left(t\right)+\frac{j}{\pi t}\right)*u_{k}\left(t\right)\right]\cdot e^{-j\omega_{k}t}∥}_{2}^{2}+{∥x\left(t\right)-\sum_{k=1}^{K}u_{k}\left(t\right)∥}_{2}^{2}\\ \;\; & +\langle \lambda \left(t\right),x\left(t\right)-\sum_{k=1}^{K}u_{k}\left(t\right)\rangle \end{array},$$

(12) $$u_{k}^{n+1}\left(t\right)=\underset{u_{k}\in X}{\mathrm{argmin}}\left\{\alpha {∥\partial_{t}\left[\left(\delta \left(t\right)+\frac{j}{\pi t}\right)*u_{k}\left(t\right)\right]\cdot e^{-j\omega_{k}t}∥}_{2}^{2}+{∥x\left(t\right)-\sum_{i}u_{i}\left(t\right)+\frac{\lambda (t)}{2}∥}_{2}^{2}\right\}.$$

Alternate direction method of multipliers (ADMM) is used to solve the unconstrained variational problem, and the saddle point is obtained by updating $u_{k}^{n+1}$, $\omega_{k}^{n+1}$, and $\lambda^{n+1}$ in frequency respectively. The specific formula is denoted as Equations (13)–(15). Where component $u_{k}^{n+1}\left(t\right)$ can be expressed as Equation (12).

(13) $${\hat{u}}_{k}^{n+1}\left(\omega \right)=\left[\hat{x}\left(\omega \right)-\sum_{i\neq 1}{\hat{u}}_{i}\left(\omega \right)+\frac{\hat{\lambda }\left(\omega \right)}{2}\right]\cdot \frac{1}{1+2\alpha {\left(\omega -\omega_{k}\right)}^{2}}$$

(14) $$\omega_{k}^{n+1}=\frac{\int_{0}^{\infty }\omega {\left|{\hat{u}}_{k}\left(\omega \right)\right|}^{2}d\omega }{\int_{0}^{\infty }{\left|{\hat{u}}_{k}\left(\omega \right)\right|}^{2}d\omega }$$

(15) $${\hat{\lambda }}^{n+1}\left(\omega \right)={\hat{\lambda }}^{n}\left(\omega \right)+\tau \left[\hat{x}\left(\omega \right)-\sum_{k}{\hat{u}}_{k}^{n+1}\left(\omega \right)\right],$$

where ${}^{\wedge }$ represents the Fourier transform, *n* is the number of iterations, and τ denotes the parameter of noise tolerance. Finally, according to Equations (13)–(15), $u_{k}^{n+1}$, $\omega_{k}^{n+1}$, and $\lambda^{n+1}$ are continuously updated until the iteration termination condition is met. The iteration termination condition is shown in Equation (16):

(16) $$\frac{\sum_{k}{∥{\hat{u}}_{k}^{n+1}-{\hat{u}}_{k}^{n}∥}_{2}^{2}}{{∥{\hat{u}}_{k}^{n}∥}_{2}^{2}}<e.$$

In conclusion, the detailed steps of the VMD algorithm are shown in Algorithm 2.
**Algorithm 2** VMD algorithm1:Initialize $\left\{{\hat{u}}_{k}\right\},\left\{w_{k}\right\},\left\{\hat{\lambda }\right\}$ and *n*.2:Update ${\hat{u}}_{k}^{n+1}\left(\omega \right)$ by Equation (13).3:Update $\omega_{k}^{n+1}$ by Equation (14).4:Update ${\hat{\lambda }}^{n+1}\left(\omega \right)$ by Equation (15).5:Repeat the above updating steps until the iteration termination condition Equation (16) is met.

## 3. Methodology

This section will first introduce the concept of VMD parameter optimization. Then the fitness function based on permutation entropy is explained. Next, a new method based on Hausdorff distance (HD) between the probability density function (PDF) of all BLIMFs and that of the original signal is proposed to determine the relevant modes. Finally, the detailed steps of the proposed method are given.

### 3.1. VMD Parameter Optimization

The VMD algorithm is not a recursive signal decomposition method, and it can adaptively decompose the signal in frequency domain and effectively get the BLIMFs of the signal. Compared with the EMD algorithm, the VMD algorithm has a clearer mathematical theory and better noise robustness [32]. Because of the advantages of the VMD algorithm, the VMD algorithm is widely used in many fields [33,34,35].

However, some studies have proved that the data processing results by using the VMD method are affected by quadratic penalty factors α and the number of decomposition mode *K* [36]. As for the selection of two parameters, the traditional selection method is depending on experience [29]. For the sake of better determining the combination of parameters, this paper proposes a parameter optimization method based on the BSAS algorithm and permutation entropy.

### 3.2. Fitness Function Based on Permutation Entropy

In order to optimize the two parameters *K* and α of VMD by using BSAS algorithm, the optimization index (i.e., the fitness function) should be determined first.

Permutation entropy (PE) is proposed by Bandt et al., and it is a method to judge the randomness and complexity of time series [37]. It has small calculation and strong anti-interference ability, and it is applied in many fields as fitness function or feature selection [38,39]. The specific introduction of PE is as follows:

The matrix shown in Equation (17) is the phase space reconstruction of time series {x(i),i=1,2,⋯,n}.

(17) $$\begin{array}{cc} \; & \left[\begin{array}{ccc} x(1) & x(1+\tau ) & \cdots x(1+(m-1)\tau )\\ x(2) & x(2+\tau ) & \cdots x(2+(m-1)\tau )\\ x(j) & x(j+\tau ) & \cdots x(j+(m-1)\tau )\\ \cdots & \cdots & \cdots \\ x(K) & x(K+\tau ) & \cdots x(K+(m-1)\tau ) \end{array}\right] \end{array},$$

where *m* and τ are embedding dimension and delay time respectively, and j=1,2,3,…,K. Each row in the matrix represents a reconstruction component, so there are *K* reconstruction components in total, K=n−(m−1)τ.

The *j*th component (x(j),x(j+τ),…,x(j+(m−1)τ) in the reconstruction matrix is rearranged in ascending order, as shown in Equation (18). $j_{1},j_{2},\ldots ,j_{m}$ represents the index number of each element in the reconstruction component.

(18) $$x\left(i+\left(j_{1}-1\right)\tau \right)\leq x\left(i+\left(j_{2}-1\right)\tau \right)\leq \ldots \leq x\left(i+\left(j_{m}-1\right)\tau \right).$$

If there are two identical reconstruction components, for example:

(19) $$x\left(i-\left(j_{p}-1\right)\tau \right)=x\left(i-\left(j_{q}-1\right)\tau \right).$$

Then it should be sorted by the size of the values of $j_{p}$ and $j_{q}$. Therefore, for any vector x(i) composed of time series data, a set of symbol sequences can be obtained:

(20) $$S\left(l\right)=\left(j_{1},j_{2},\cdots ,j_{m}\right).$$

It should be noted that *m* dimensional phase space has m! different sequence of symbols, and S(l) is one of them. If the probability of each symbol sequence is calculated as $P_{1},P_{2},\ldots ,P_{k}$, then the permutation entropy of time sequence x(i) can be defined as Equation (21):

(21) $$H_{P}=-\sum_{j=1}^{k}P_{j}\ln P_{j}.$$

The value of $H_{P}$ indicates the randomness of the time series x(i). The smaller the value of $H_{P}$ is, the more orderly it is; otherwise, the more random it is. It means that after the original signal is decomposed by VMD algorithm, the less noise the BLIMF component contains, the less PE it has.

VMD algorithm is used to decompose the original signal in each iteration. The permutation entropy of each BLIMF component is calculated, where the minimum value is taken as the optimal result of the current iteration. In the optimization process, the fitness function is given as follows:

(22) $$f\left(K,\alpha \right)=\min\left\{H_{p}\left(BLIMFs\right)\right\}.$$

### 3.3. Selecting Relevant Modes

According to the principle of the VMD algorithm mentioned above, the original signal can be decomposed into several BLIMF components from low to high frequency by using the VMD algorithm. Generally speaking, the random noise is mainly concentrated in the high frequency component. In order to reduce the noise of the original signal better by using the VMD algorithm, it is necessary to find the demarcation point between the noise BLIMF component and the noiseless BLIMF component, which also means to determine the relevant modes.

In traditional VMD noise reduction method, the demarcation point is determined by calculating the continuous mean square error (CMSE) of each BLIMFs. Where the BLIMF with the first minimum value of CMSE is the demarcation point $k_{\mathrm{th}}$ [40]. The calculation formula of CMSE and the definition of demarcation point are shown in Equations (23) and (24). Where $u_{k}\left(t\right)$ is the component obtained by using VMD algorithm to decompose the original signal, and ${\hat{x}}_{k}$ is the reconstructed signal.

(23) $$\begin{array}{cc} \mathrm{CMSE}\left({\hat{x}}_{k},{\hat{x}}_{k+1}\right) & =\frac{1}{N}\sum_{i=1}^{N}{\left[{\hat{x}}_{k}\left(t_{i}\right)-{\hat{x}}_{k+1}\left(t_{i}\right)\right]}^{2}\\ \; & =\frac{1}{N}\sum_{i=1}^{N}{\left[u_{k}\left(t_{i}\right)\right]}^{2}, \end{array}$$

(24) $$k_{\mathrm{th}}=\underset{1\leq k\leq N-1}{\mathrm{argmin}}\left[\mathrm{CMSE}\left({\hat{x}}_{k},{\hat{x}}_{k+1}\right)\right].$$

However, the effect of this method is often not ideal, and it greatly reduces the noise reduction effect of the VMD algorithm [13,20]. In this paper, the Hausdorff distance (HD) between the probability density function (PDF) of all BLIMFs and that of the signal to be decomposed is proposed to determine the demarcation point.

HD was proposed by Huttenlocher in 1993 [41]. It is often used to calculate the similarity of two different sample sets. In this paper, HD is utilized to calculate the similarity between each BLIMFs and the original signal.

According to the principle of HD, the HD of two sample sets A=$\left\{a_{1},\cdots ,a_{i}\right\}$ and B=$\left\{b_{1},\cdots ,b_{j}\right\}$ are as follows:

(25) HD(A,B)=max{hd(A,B),hd(B,A)}.

Equation (25) indicates bidirectional Hausdorff distance between $A=\left\{a_{1},\cdots ,a_{i}\right\}$ and $B=\left\{b_{1},\cdots ,b_{j}\right\}$, and it is the basic formula of HD. Where hd(A,B) and hd(B,A) are called unidirectional Hausdorff distance of sets A to B and B to A, respectively, as shown below:

(26) $$hd\left(A,B\right)=\max_{a\in A}\min_{b\in B}∥a-b∥$$

(27) $$hd\left(B,A\right)=\max_{b\in B}\min_{a\in A}∥b-a∥.$$

In Equations (26) and (27), *a* and *b* represent elements in sets A and B respectively, and ∥·∥ refers to l2-norm between two elements.

The probability density function (PDF) can represent the distribution of data, and it can also be used as a similarity measure for different data [42]. Therefore it can be combined with HD to calculate the similarity for signals, as follows:

(28) $$S\left(i\right)=\mathrm{HD}\left[PDF\left(x\left(t\right)\right),PDF\left(BLIMFi\right)\right]$$

As shown in Equation (28), where S(i) is HD based PDF between each BLIMFs and the original signal x(t).

To sum up, the selection of relevant modes (i.e., components determined by the demarcation point), can be solved by Equation (29):

(29) $$k_{\mathrm{th}}=\underset{1\leq i\leq K}{\arg\max}\{∥S\left(i+1\right)-S\left(i\right)∥\},$$

where $k_{\mathrm{th}}$ is the demarcation point, and it also represents the maximum slope based on HD of the PDF for two adjacent BLIMF components [43]. *i* represents the *i*th BLIMF, and there are *K* in total.

### 3.4. Proposed Methodology

According to the above analysis and statement, the detailed steps of the proposed method can be described as follows:

Step 1: The parameters of the BSAS algorithm are initialized. At the same time, the parameters in permutation entropy are initialized too.
Step 2: Firstly, the VMD algorithm is used to decompose the original signal. After that, the fitness function value based on permutation entropy is calculated, and the parameters *K* and α are optimized by using the BSAS algorithm.
Step 3: Determine whether the termination condition is met. If it is, optimal parameter combination (K,α) is saved, otherwise step 2 is repeated.
Step 4: The original signal is decomposed by using the VMD algorithm based on optimal parameter combination.
Step 5: The HD between the PDF of all BLIMF and that of the original signal is calculated to determine the demarcation point.
Step 6: According to the demarcation point, the selected BLIMF components are retained as the relevant modes, and the unselected BLIMF components are removed.
Step 7: The relevant modes are reconstructed, and finally the denoised signal is obtained.

**Figure 1:**
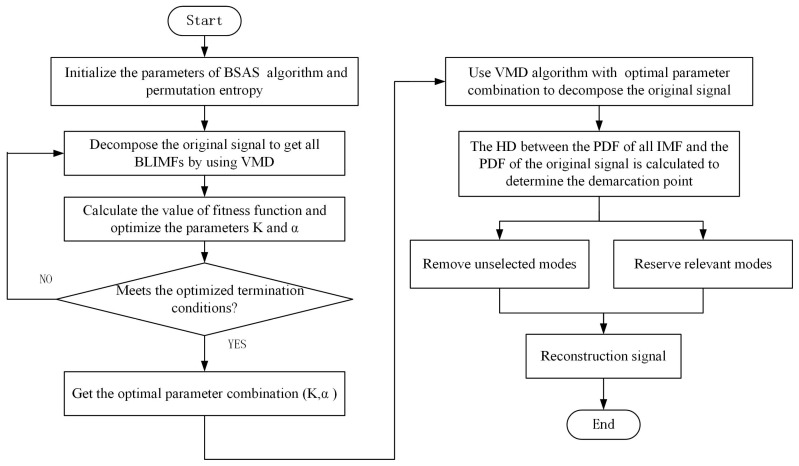
Flowchart of the proposed method.

Based on the above introduction, the flowchart of the proposed scheme is shown in Figure 1. It should be pointed out that the algorithm proposed in this paper mainly includes two parts, one is the parameter optimization of the VMD algorithm, the other is the decomposition and reconstruction of signals. Due to the large amount of calculation in the process of parameter optimization, this part usually needs to be processed offline. However, the proposed method is still superior to the traditional trial and error method in terms of result reliability and time consumption. Therefore, this paper will mainly study the effect of noise reduction, and not do too much analysis on the timeliness and delay analysis of the algorithm.

## 4. Simulation and Analysis

In this section, the performance of the proposed scheme is demonstrated by simulation. Firstly, the simulation environment is introduced, which including initialization of algorithm parameters and selection of simulation signals. After that, the simulation results are analyzed and compared with the results of several common denoising schemes.

### 4.1. Simulation Environment

**Figure 2:**
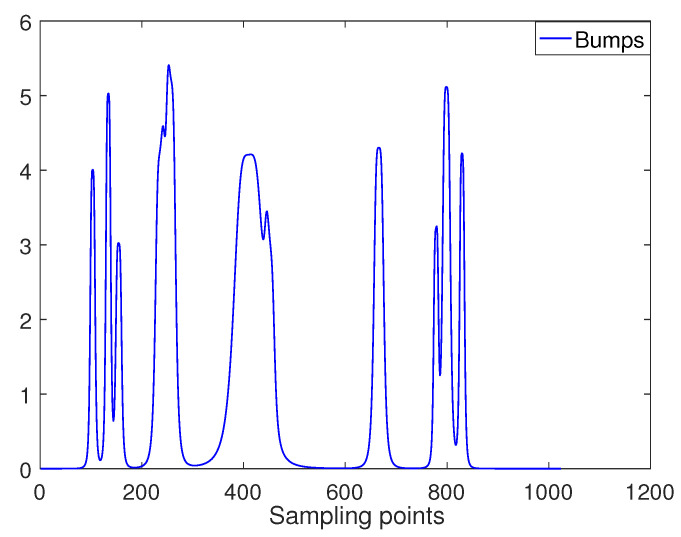
Bumps original signal.

Bumps signal is a kind of continuously changing signal, which has a wide range of frequency coverage. It is similar to the characteristics of the FOG signal. The original Bumps signal is shown in Figure 2.

**Figure 3:**
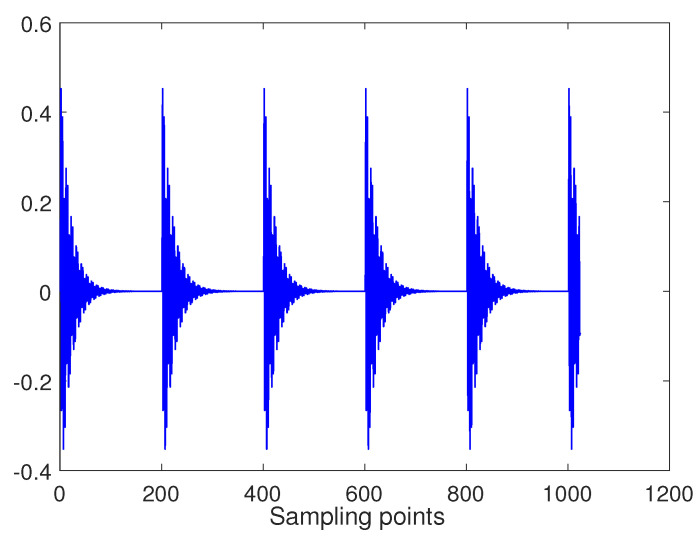
Impulse signal.

**Figure 4:**
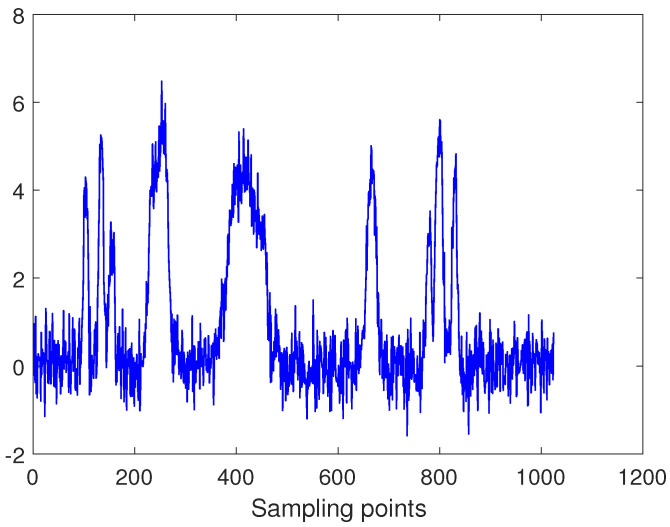
Simulation signal.

In practice, the FOG signal is often accompanied by some small vibration or bumpy signal and a large number of random noise in actual use. So in order to better verify the performance of the proposed algorithm, the impulse signal, as shown in Figure 3, is added to the Bumps signal. In addition, Gaussian white noise is also added to the whole signal, where SNR = 10 dB and signal length N = 1024. The final simulation signal is as shown in Figure 4.

Besides, some parameters of the proposed algorithm need to be set before the simulation. To begin with, the parameters of the BSAS algorithm are initialized as follows—the number of beetles is 10 and the maximum number of iterations is 50; The initial value of step size and sensing distance are 5 and 4 respectively; The decay coefficients of step and sensing distance are both 0.95. The probability constants of position updating and step updating are both set to 0.85, and the maximum number of invalid search is 2. Then, according to experience, the embedding dimension of permutation entropy is set to 6, and the delay time is initialized to 1.

### 4.2. Simulation Results and Analysis

**Figure 5:**
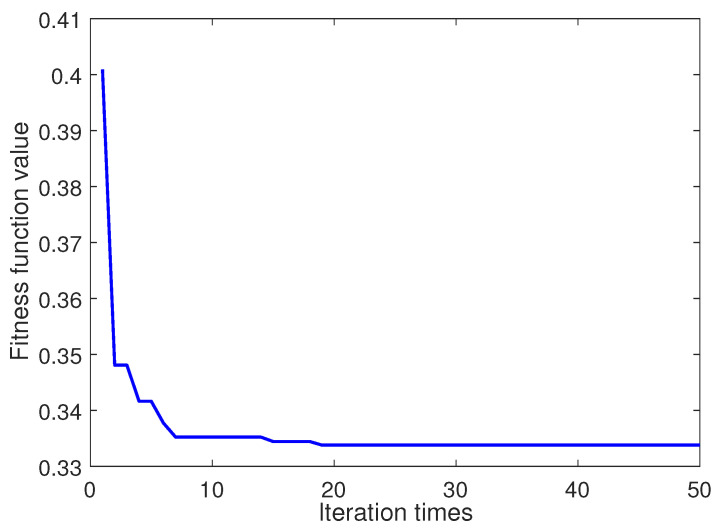
Convergence curve of the beetle swarm antenna search (BSAS) algorithm.

According to the steps of the proposed algorithm, firstly, two parameters *K* and α of VMD are optimized by the BSAS algorithm. The convergence curve based on the BSAS algorithm is shown in Figure 5. In the first 10 iterations, the speed of curve convergence is the fastest. After that, the convergence tends to be stable.

It can be seen from the optimization process that the minimum value of entropy permutation is 0.3338, and the corresponding *K* and α are 7 and 1530 respectively.

**Figure 6:**
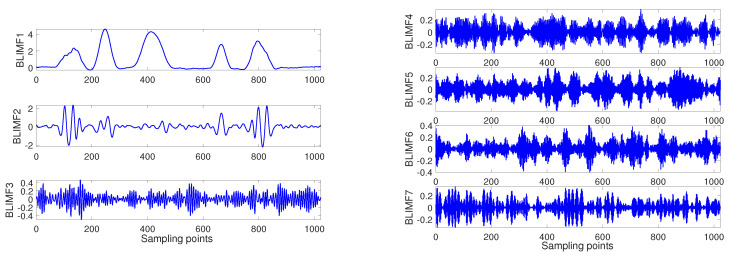
Decomposition result of the simulation signal.

After that, the simulation signal is decomposed by VMD algorithm based on the optimal parameter combination which obtained by BSAS algorithm, and all BLIMFs are as shown in the Figure 6:

Then, according to step 5 of the proposed algorithm, the Hausdorff distance between the PDF of all BLIMFs and that of the original signal should be calculated to determine the demarcation point.

**Figure 7:**
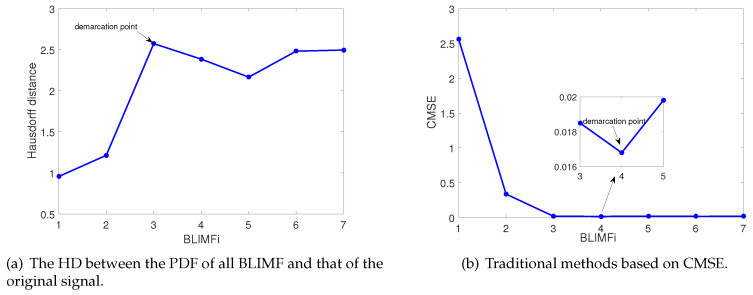
Determination of demarcation point.

As shown in Figure 7a, in the light of the definition of demarcation point proposed by Equations (28) and (29), on the one hand, BLIMF1 and BLIMF2 are reserved as relevant modes for signal reconstruction. On the other hand, BLIMF3 to BLIMF7 are removed as unselected modes. Figure 7b shows the result of traditional scheme based on CMSE.

Finally, the selected components are reconstructed to get the denoised signal. In order to evaluate the performance of the proposed algorithm, two evaluation indexes, signal-to-noise ratio (SNR) and root mean square error (RMSE), are given as Equations (30) and (31).

(30) $$\mathrm{SNR}=10\cdot \log\frac{\sum_{i=1}^{N}{\left|x\left(i\right)\right|}^{2}}{\sum_{i=1}^{N}{\left|\hat{x}\left(i\right)-x\left(i\right)\right|}^{2}},$$

(31) $$\mathrm{RMSE}=\sqrt{\frac{\sum_{i=1}^{N}{\left|\hat{x}\left(i\right)-x_{model}\left(i\right)\right|}^{2}}{N},}$$

where *N* is the length of the signal. *x* is the noiseless signal, $\hat{x}$ is the denoised signal, and $x_{model}$ represents ideal value.

**Figure 8:**
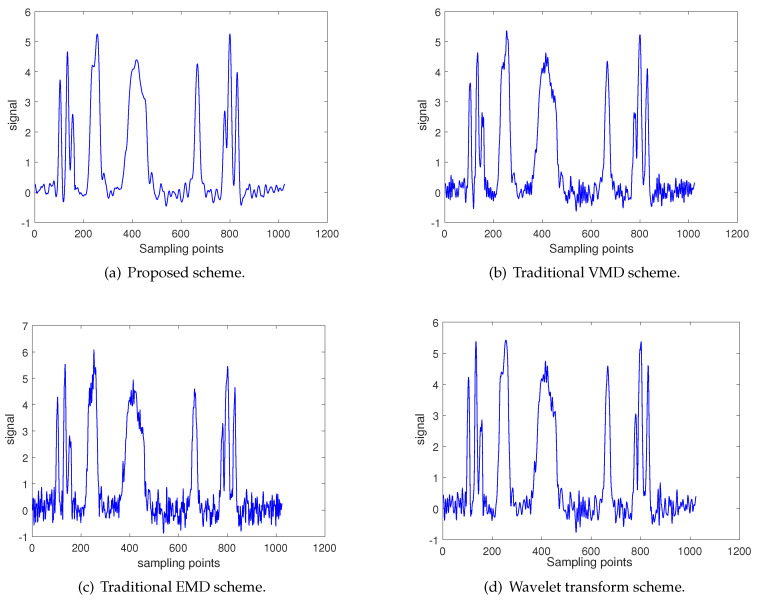
Denoised results.

Figure 8a shows the noise reduction results based on the proposed scheme. At the same time, three other common denoising methods, including the traditional VMD scheme, the traditional EMD scheme and the scheme based on wavelet transformation (WT), are also used to process the simulation signals. As shown in Figure 8, the noise reduction effect is worst by using the traditional EMD method, and it is obvious that a lot of noise exists in its results. The SNR and RMSE of this method are 15.2305 and 0.3116 respectively. The noise reduction effect based on WT method and traditional VMD method is satisfactory, but both of them have some small noise signals. The SNR and RMSE of the two methods are given in Table 1. The best dnoised effect is obtained by the proposed scheme, which has the smoothest and most stable noise reduction result curve. Among all methods, the proposed method has the largest SNR and the smallest RMSE, which are 18.3232 and 0.2183 respectively.

Based on the Figure 8 and two evaluation indexes shown in Table 1, it can be concluded that after processing the original simulation signal with the proposed scheme, the denoised signal has the highest accuracy and the best stability. Therefore, the proposed scheme is superior to the other three schemes in noise reduction performance.

## 5. Experimental Analysis

**Figure 9:**
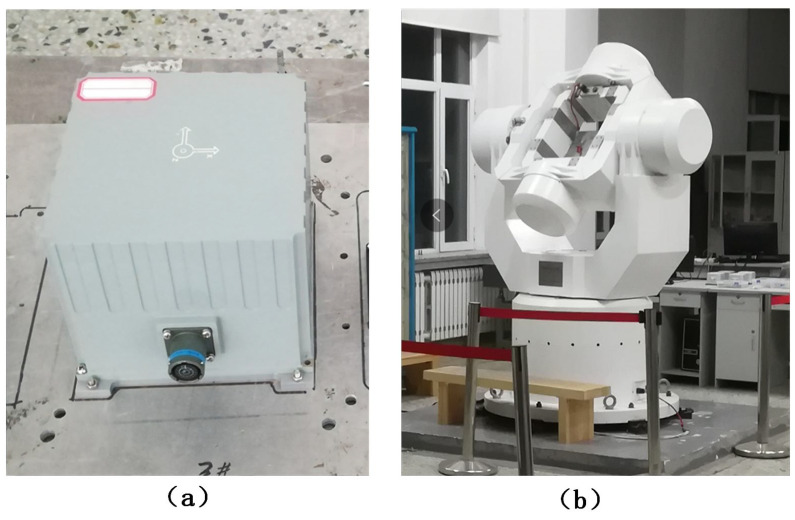
Experimental equipment. (**a**) FOG-based IMU. (**b**) Three-axis turntable.

In this section, static test and dynamic rotation test experiments based on FOG are carried out respectively to prove the feasibility of the proposed method. The equipment which will be used in experiments is a high precision three-axis turntable, as shown in Figure 9b. In the experiment, the temperature of room was kept at 26 degrees centigrade.

It is worth noting that the FOG-based inertial measurement unit (IMU) used in two experiments is independently designed and produced by Harbin Engineering University. The parameters of the FOG are given in Table 2.

### 5.1. Static Test Experiment

**Figure 10:**
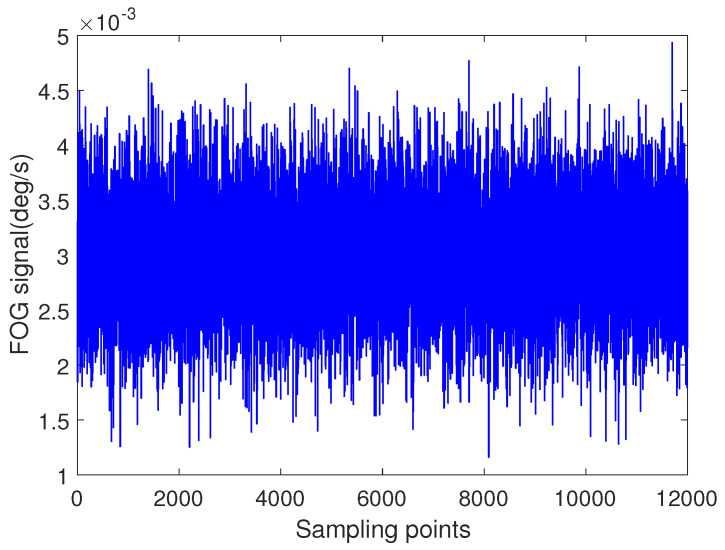
Fiber optic gyroscope (FOG) static test raw signal.

In the static test experiment, the FOG is placed on a three-axis turntable firstly. Then, the signal of measurement axis, which is aligned with the upward direction, is collected. The sampling frequency is 100 Hz. In order to avoid the measurement error of the instrument in the start-up stage, 12,000 sampling points are selected in the middle stage of the whole measurement process, as shown in Figure 10.

**Figure 11:**
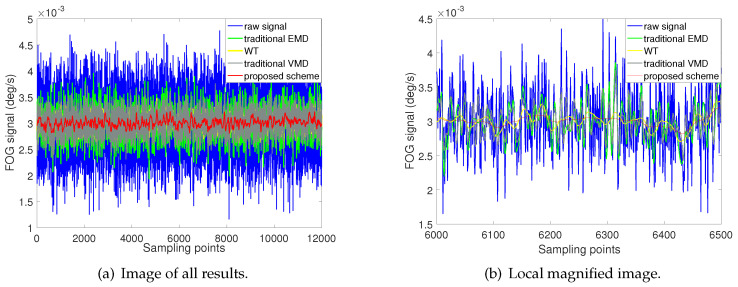
Denoising results of four different methods for static test experiment.

The raw signals are denoised by the proposed scheme, and the other three schemes are also implemented separately. The results of the four methods are shown in Figure 11.

It can be seen from Figure 11a that all four methods can effectively reduce the noise of FOG static signal. Figure 11b is a local enlarged graphs, which shows part of the noise reduction result. The scheme proposed in this paper is represented by red curve, which has the best effect and stability in noise reduction performance. The noise reduction effect of the WT method and the traditional VMD method are the second and third respectively, and their results are similar. Although the traditional EMD method, represented by green curve, can also reduce the noise of the original signal, its effect is the worst compared with the other three methods.

In order to better compare the performance of different methods, in addition to the RMSE, the noise intensity (NI) [44] is proposed as an evaluation index for all experiments. The definition of NI is similar to the standard deviation, which can be expressed as follows:

(32) $$\mathrm{NI}=\sqrt{\frac{\sum_{i=1}^{N}{\left({\hat{x}}_{i}-\bar{x}\right)}^{2}}{N}}.$$

Table 3 records the noise intensity and root mean square error for four method. The minimum value of the root mean square error comes from the proposed method, which is 1.2939 $\times 10^{-4}$${}^{\circ }$/s. For the other three methods, the root mean square error is 2.2630 $\times 10^{-4}$${}^{\circ }$/s, 2.8105$\times 10^{-4}$${}^{\circ }$/s and 1.6716$\times 10^{-4}$${}^{\circ }$/s respectively. In terms of the noise intensity, the proposed scheme is 7.3872$\times 10^{-5}$${}^{\circ }$/s, which is also the minimum in the four methods. The maximum noise intensity obtained by traditional EMD method is 2.6109$\times 10^{-4}$${}^{\circ }$/s. The noise intensity of traditional VMD method and WT method are 1.9982$\times 10^{-4}$${}^{\circ }$/s and 1.2901$\times 10^{-4}$${}^{\circ }$/s respectively.

In summary, the static test experiment demonstrate that the proposed scheme can effectively reduce the noise of the FOG static signal. In terms of noise reduction effect, the proposed scheme is better than the other three schemes.

### 5.2. Dynamic Rotation Test Experiment

In order to further verify the feasibility of the proposed scheme, a dynamic rotation test experiment is designed in this paper. First of all, the FOG-based IMU is placed on the three-axis turntable, and its measuring axis is aligned with the upward direction. After that, the FOG is rotated around the measuring axis by using the three-axis turntable, and the rotation speed is 5${}^{\circ }$/s and 10${}^{\circ }$/s respectively. As the same as the static test, 12,000 sampling points are selected for processing.

**Figure 12:**
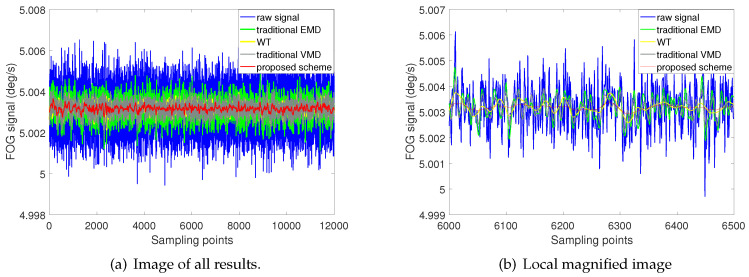
Denoising results of four different methods at a rotational speed of 5 ${}^{\circ }$/s.

**Figure 13:**
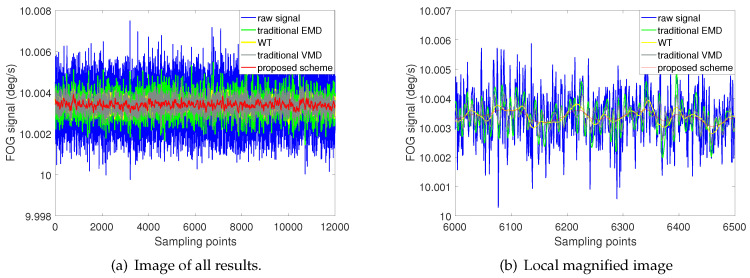
Denoising results of four different methods at a rotational speed of 10 ${}^{\circ }$/s.

Figure 12 and Figure 13 show the noise reduction results of the four methods respectively at rotation speeds of 5${}^{\circ }$/s and 10${}^{\circ }$/s. Two groups of experimental results show that the noise reduction effect of the proposed scheme is significantly better than the other three schemes at different rotation speeds. From the the partial enlarged graphs, Figure 12b and Figure 13b, it can be seen that the results of the proposed scheme are smoother, and has the least signal burr. More information about the performance evaluation of the four schemes is shown in Table 4.

Table 4 gives the noise reduction results of four methods at different rotation speeds 5${}^{\circ }$/s and 10${}^{\circ }$/s. It can be seen from the table that the NI of the proposed method is 1.2759$\times 10^{-4}$${}^{\circ }$/s and 1.3437$\times 10^{-4}$${}^{\circ }$/s respectively, and RMSE is 1.4448$\times 10^{-4}$${}^{\circ }$/s and 1.8412$\times 10^{-4}$${}^{\circ }$/s, which are all the minimum values compared with other methods. The results of traditional EMD method are the worst. NI and RMSE are 4.8796$\times 10^{-4}$${}^{\circ }$/s and 4.9211$\times 10^{-4}$${}^{\circ }$/s respectively in 5${}^{\circ }$/s; 4.9692$\times 10^{-4}$${}^{\circ }$/s and 5.1202$\times 10^{-4}$${}^{\circ }$/s respectively in 10${}^{\circ }$/s.

From the processing results of the original signal at different rotation speeds above, it can be concluded that in different test environments, the proposed scheme in this paper always has better noise reduction performance.

## 6. Conclusions

This paper presents a new method for the denoising of a FOG signal. This method is based on the BSAS algorithm and permutation entropy to optimize two parameters *K* and α of the VMD algorithm firstly. Where the fitness function is designed by calculating the permutation entropy of each BLIMF. After that, in order to better find the relevant modes, the Hausdorff distance between the probability density function of all BLIMFs and that of the original signal is proposed in this paper to determine the demarcation point. Finally, the selected modes are reconstructed into denoised signal.

For the sake of demonstrating the performance of proposed method, simulations and experiments are performed in this paper. The simulation results show that the proposed scheme is superior to the traditional VMD method, the traditional EMD method and the WT method in terms of noise reduction effect and stability. In addition, the feasibility of proposed scheme is further proved by static test experiment and dynamic rotation test experiment.

However, the method proposed in this paper is still in the stage of development and exploration. Due to the large amount of calculation in the process of optimization, it cannot be well adapted to online application. Therefore, the future work will focus on the improvement of the new algorithm to achieve online noise reduction.

## Figures and Tables

**Table 1:** Noise reduction performance index of four methods.

Method	Signal-to-Noise Ratio (SNR/dB)	Root Mean Square Error (RMSE)
Proposed method	18.3232	0.2183
Traditional VMD	17.2877	0.2459
Traditional EMD	15.2305	0.3116
Wavelet transform	17.3686	0.2436

**Table 2:** Parameters of the FOG.

Parameter Item	Parameter Values
FOG dynamic range (${}^{\circ }$/s)	≤600
FOG bias stability (${}^{\circ }$/h)	0.03
FOG random bias (${}^{\circ }/h^{1/2}$)	0.003

**Table 3:** Noise intensity (NI) and root mean square error (RMSE) comparison for the four methods.

Method	Noise Intensity (NI)	Root Mean Square Error (RMSE)
Proposed method	7.3872$\times 10^{-5}$	1.2939$\times 10^{-4}$
Traditional VMD	1.9982$\times 10^{-4}$	2.2630$\times 10^{-4}$
Traditional EMD	2.6109$\times 10^{-4}$	2.8105$\times 10^{-4}$
Wavelet transform	1.2901$\times 10^{-4}$	1.6716$\times 10^{-4}$

**Table 4:** Denoising results of four methods at different rotation speed.

Rotational Speed	Proposed Method		Traditional VMD		Traditional EMD		Wavelet Transform	
	NI	RMSE	NI	RMSE	NI	RMSE	NI	RMSE
5 (${}^{\circ }$/s)	1.2759$\times 10^{-4}$	1.4448$\times 10^{-4}$	3.2013$\times 10^{-4}$	3.2723$\times 10^{-4}$	4.8796$\times 10^{-4}$	4.9211$\times 10^{-4}$	2.3837$\times 10^{-4}$	2.4785$\times 10^{-4}$
10 (${}^{\circ }$/s)	1.3437$\times 10^{-4}$	1.8412$\times 10^{-4}$	3.2248$\times 10^{-4}$	3.4618$\times 10^{-4}$	4.9692$\times 10^{-4}$	5.1202$\times 10^{-4}$	2.3852$\times 10^{-4}$	2.6971$\times 10^{-4}$
